# Minigene Splicing Assays Identify 12 Spliceogenic Variants of *BRCA2* Exons 14 and 15

**DOI:** 10.3389/fgene.2019.00503

**Published:** 2019-05-28

**Authors:** Eugenia Fraile-Bethencourt, Alberto Valenzuela-Palomo, Beatriz Díez-Gómez, María José Caloca, Susana Gómez-Barrero, Eladio A. Velasco

**Affiliations:** ^1^Splicing and Genetic Susceptibility to Cancer, Instituto de Biología y Genética Molecular (CSIC-UVa), Valladolid, Spain; ^2^Instituto de Biología y Genética Molecular (CSIC-UVa), Valladolid, Spain; ^3^VISAVET-Universidad Complutense de Madrid, Madrid, Spain

**Keywords:** breast cancer, BRCA2, DNA variants, splicing, hybrid minigenes

## Abstract

A relevant fraction of *BRCA2* variants is associated with splicing alterations and with an increased risk of hereditary breast and ovarian cancer (HBOC). In this work, we have carried out a thorough study of variants from BRCA2 exons 14 and 15 reported at mutation databases. A total of 294 variants from exons 14 and 15 and flanking intronic sequences were analyzed with the online splicing tools NNSplice and Human Splicing Finder. Fifty-three out of these 294 variants were selected as candidate splicing variants. All variants but one, were introduced into the minigene MGBR2_ex14-20 (with exons 14–20) by site-directed mutagenesis and assayed in MCF-7 cells. Twelve of the remaining 52 variants (23.1%) impaired splicing at different degrees, yielding from 5 to 100% of aberrant transcripts. Nine variants affected the natural acceptor or donor sites of both exons and three affected putative enhancers or silencers. Fluorescent capillary electrophoresis revealed at least 10 different anomalous transcripts: 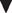
(E14q5), Δ (E14p10), Δ(E14p246), Δ(E14q256), Δ(E14), Δ(E15p12), Δ(E15p13), Δ(E15p83), Δ(E15) and a 942-nt fragment of unknown structure. All transcripts, except for Δ(E14q256) and Δ(E15p12), are expected to truncate the BRCA2 protein. Nine variants induced severe splicing aberrations with more than 90% of abnormal transcripts. Thus, according to the guidelines of the American College of Medical Genetics and Genomics, eight variants should be classified as pathogenic (c.7008-2A > T, c.7008-1G > A, c.7435+1G > C, c.7436-2A > T, c.7436-2A > G, c.7617+1G > A, c.7617+1G > T, and c.7617+2T > G), one as likely pathogenic (c.7008-3C > G) and three remain as variants of uncertain clinical significance or VUS (c.7177A > G, c.7447A > G and c.7501C > T). In conclusion, functional assays by minigenes constitute a valuable strategy to primarily check the splicing impact of DNA variants and their clinical interpretation. While bioinformatics predictions of splice site variants were accurate, those of enhancer or silencer variants were poor (only 3/23 spliceogenic variants) which showed weak impacts on splicing (∼5–16% of aberrant isoforms). So, the Exonic Splicing Enhancer and Silencer (ESE and ESS, respectively) prediction algorithms require further improvement.

## Introduction

Since the discovery of the breast cancer genes *BRCA1* (OMIM #113705) and *BRCA2* (OMIM #600185) (Miki et al., 1994; Wooster et al., 1995), nearly 17,000 different variants of both genes have been recorded at the ClinVar database^1^ (date last accessed; November 2018). Germline inactivating variants in *BRCA1* and *BRCA2* confer high lifetime risks of breast and ovarian cancers (Mavaddat et al., 2013). Also, other cancer types, such as prostate, pancreatic and melanoma, are associated with pathogenic variants in these genes (Petrucelli et al., 2013). Despite the high penetrance of *BRCA* pathogenic variants, they are responsible for only ∼15–20% of hereditary breast and ovarian cancer (HBOC) (Stratton and Rahman, 2008). In fact, HBOC is a highly genetically heterogeneous disease with about 25 known or proposed susceptibility genes (Nielsen et al., 2016). Apart from the BRCA genes, *PALB2* (OMIM #610355), *ATM* (OMIM #607585), and *CHEK2* (OMIM #604373) have a prominent contribution since, in a recent study, more than 30% of pathogenic variants were found in these genes (Buys et al., 2017).

Commonly, the variants are classified attending to their predicted effect on the protein so that truncating variants (frameshift and nonsense) are directly classified as pathogenic, while intronic, missense and synonymous variants are usually considered to be variants of uncertain clinical significance (VUS). In fact, VUS are identified by a relevant proportion of BRCA genetic tests (∼20%), which hamper genetic counseling and subsequent preventive or therapeutic actions, since risk assessment is solely based on family history (Radice et al., 2011; Eccles et al., 2015; Ricks et al., 2015).

Furthermore, other upstream gene-expression processes, such as transcription or splicing, can be impaired if regulatory motifs are targeted by nucleotide variations (Wang and Cooper, 2007). Splicing is the process by which introns are removed from a pre-mRNA and exons are consecutively joined. This mechanism is performed in the nucleus by the spliceosome, a macrocomplex constituted by 5 small nuclear ribonucleoproteins (snRNPs) and many other associated proteins (De Conti et al., 2012). The spliceosome recognizes in the pre-mRNA specific sequences which define the exons/introns boundaries and other elements needed to carry out the process. These sequences are: the acceptor or 3′ splice site (3′ss), the donor or 5′ splice site (5′ss), the branch point, the polypyrimidine tract and the auxiliary *cis* sequences known as splicing regulatory elements (SREs) where enhancer or silencer *trans* factors can bind. Therefore, any change in the sequence may disrupt splicing (Cartegni et al., 2002). Splicing variants usually break the 3′ss or 5′ss leading to abnormal splicing events such as exon skipping, alternative site usage or intron retention. However, they may also create new splicing sites or strengthen cryptic ones that would then be recognized. Other mechanism that may alter splicing is the disruption of exonic/intronic splicing enhancers (ESEs/ISEs) or the creation of exonic/intronic splicing silencers (ESSs/ISSs) (Abramowicz and Gos, 2018). Nevertheless, it is extremely difficult to identify active SREs and predict the impact of the DNA variants on splicing given the low accuracy of SRE-detection softwares. Therefore, splicing variants can induce abnormal transcripts that either introduce premature termination codons (PTC), in-frame loss of essential protein domains or even inclusion of new translated sequences. Consequently, variants with impact on splicing (or *spliceogenic* variants) may be associated with an increased risk of a given disease. This ethiopathogenic mechanism has been so far underestimated, even though some authors have suggested that spliceogenic variants may represent more than 60% of disease-causing mutations (López-Bigas et al., 2005).

Previous studies have shown that a significant number of splicing variants have been detected in *BRCA2* (Spurdle et al., 2008; Rebbeck et al., 2018). In fact, previous results from our group showed that more than 50% of tested variants of *BRCA2* exons 16–27 impaired splicing (Acedo et al., 2012, 2015; Fraile-Bethencourt et al., 2017, 2018). Likewise, at least 24 different BRCA2 alternative transcripts have been identified. They are helpful to interpret the splicing outcomes of genetic variations (Fackenthal et al., 2016) and suggest a fine regulation of *BRCA2* exon processing. This feature is supported by the fact that several ESE-rich regions have been functionally mapped by exonic deletions throughout most *BRCA2* exons. Thus, these motifs would be involved in precise exon recognition and alternative splicing events (Acedo et al., 2015; Fraile-Bethencourt et al., 2017). Moreover, we showed that functional mapping is an optimal approach that improves ESE-software predictions and facilitates the identification of spliceogenic mutations of this sort of *cis*-elements.

In this work, we have extended our analysis to *BRCA2* exons 14 and 15 by carrying out an in-depth study of candidate spliceogenic variants. We have explored the presence of splicing enhancers in exons 14 and 15 and have undertaken RNA assays of 52 selected variants from both exons.

## Materials and Methods

Ethical approval for this study was obtained from the Ethics Review Committee of the Hospital Universitario Río Hortega de Valladolid (6/11/2014).

### Bioinformatics: Databases and *in silico* Analysis

We collected *BRCA2* variants from the main databases: ClinVar^2^, the BRCA Share Database (UMD^3^) (Beroud et al., 2016) and the Breast Cancer Information Core (BIC^4^) (Supplementary Table S1). Variants and transcripts were annotated according to the Human Genome Variation Society (HGVS) guidelines on basis of the *BRCA2* GenBank sequence NM000059.1. In order to simplify, we identified transcripts with a shortened code that combines the following symbols (Lopez-Perolio et al., 2019): Δ (skipping of reference exonic sequences), 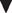 (inclusion of reference intronic sequences), E (exon), p (acceptor shift), q (donor shift). When necessary, the exact number of skipped or retained nucleotides is indicated. For example, transcript Δ(E14p10) indicates the use of an alternative acceptor site 10-nt downstream that causes a 10-nt deletion.

*In silico* analysis was made with the online softwares: NNSplice^5^ (Reese et al., 1997) and Human Splicing Finder version 3.1 (HSF^6^) that contain several prediction algorithms of different splicing motifs (Desmet et al., 2009). The following matrices were used: MaxEntScan (MES) (Yeo and Burge, 2004), the HSF branch point detection tool, ESE-finder (Cartegni et al., 2003), the HSF matrices for 9G8 and Tra2β and the HSF matrix for hnRNPA1. All the analyses were carried out with the default threshold values of NNSplice and HSF (NNSplice, 0.4; MES, 3.0; Branch point – no cut-off-; SRE (0–100 scale): SF2/ASF, 72.98; SF2/ASF (IgM – BRCA1), 70.51; SC35, 75.05; SRp40, 78.08; SRp55 73.86; 9G8, 59.245; Tra2β, 75.964 and hnRNPA1, 65.476.

### Minigene Construction and Mutations

The minigene MGBR2_14-20 was built as previously reported (Fraile-Bethencourt et al., 2017). A total of 52 variants and 8 microdeletions were introduced into the wild type (wt) minigene by site-directed mutagenesis with the QuikChange Lightning Kit (Agilent, Santa Clara, CA, United States), following the manufacturer’s instructions (Supplementary Table S2). All mutant clones were confirmed by sequencing (Macrogen, Madrid, Spain).

### MCF-7 Transfections

Approximately 2 × 10^5^ MCF-7 cells (human breast adenocarcinoma cell line) were plated in four-well plates (Nunc, Roskilde, Denmark). They were grown to 90% confluency in 0.5 mL of medium (MEME, 10% fetal bovine serum, 2 mM glutamine, 1% non-essential amino acids and 1% penicillin/streptomycin). Then, 1 μg of minigene was transfected into MCF-7 cells using low toxicity Lipofectamine (Life Technologies, Carlsbad, CA, United States) in Gibco^TM^ Opti-Mem^TM^ medium (Thermo Fisher Scientific, Waltham, MA, United States). Cells were incubated during 48h and then treated with cycloheximide 300 μg/ml (Sigma-Aldrich, St. Louis, MO, United States) for 4 h to inhibit the nonsense-mediated mRNA decay (NMD). The RNA was purified with the Genematrix Universal RNA Purification Kit (EURx, Gdańsk, Poland) with on-column DNAse I digestion.

### siRNA Assays

SR proteins were silenced in MCF7 cells by small interfering RNAs (siRNA) against the main SR proteins: SRSF1 (SF2), SRSF2 (SC35), SRSF3 (SRp20), SRSF5 (SRp40), SRSF7 (9G8), SRSF9 (SRp30c), and Tra2β (Supplementary Table S3), using anti-Luciferase siRNA as negative control. Approximately 1.5 × 10^5^ cells were subjected to a two-hit transfection in Optimem medium (Gibco – Life Technologies, Carlsbad, CA, United States) with 3 μl of Oligofectamine (Thermo Fisher Scientific) and the specific siRNA at a final concentration of 0.08 μM on day 2. Then, 2 μg of the wt minigene were transfected with low toxicity Lipofectamine (Thermo Fisher Scientific) on day 4, and RNA was extracted on day 5. Silencing was confirmed by qPCR using 10 ng of cDNA in 25 μl reaction (Supplementary Table S3). Amplification was made with SG qPCR Master Mix (Eurx, Gdańsk, Poland). Each siRNA/minigene transfection as well as all the qPCR experiments were carried out in duplicate.

### RT-PCR and Transcripts Amplification

Retrotranscription was carried out with 400 ng of RNA and the RevertAid First Strand cDNA Synthesis Kit (Life Technologies), using the specific minigene primer RTPSPL3-RV (5′-TGAGGAGTGAATTGGTCGAA-3′). Samples were incubated at 42°C for 1 h, followed by 5 min at 70°C. Transcripts were amplified with Platinum Taq DNA polymerase (Life Technologies) using 40 ng of cDNA and the primers pMAD_607FW (Patent P201231427, CSIC) and RTBR2_ex17RV2 (5′-GGCTTAGGCATCTATTAGCA-3′). PCR consisted of: denaturation step at 94°C for 2 min, followed by 35 cycles 94°C-30 s, 60°C-30 s and 72°C-1 min/kb, and a final extension step at 72°C for 5 min. Transcripts were sequenced at the Macrogen Spain facility.

In order to relatively quantify all transcripts, semi-quantitative fluorescent RT-PCRs were undertaken in triplicate with the primers pMAD_607FW (FAM-labeled) and RTBR2_ex17RV2 and Platinum Taq DNA polymerase (Life Technologies) under standard conditions except that 26 cycles were herein applied (Acedo et al., 2015). FAM-labeled products were run with LIZ-1200 Size Standard at the Macrogen facility and analyzed with the Peak Scanner software V1.0 (Life Technologies). Only peak heights ≥ 50 RFU (Relative Fluorescence Units) were considered.

## Results

### Minigene Construction and ESE Mapping

**FIGURE 1:**
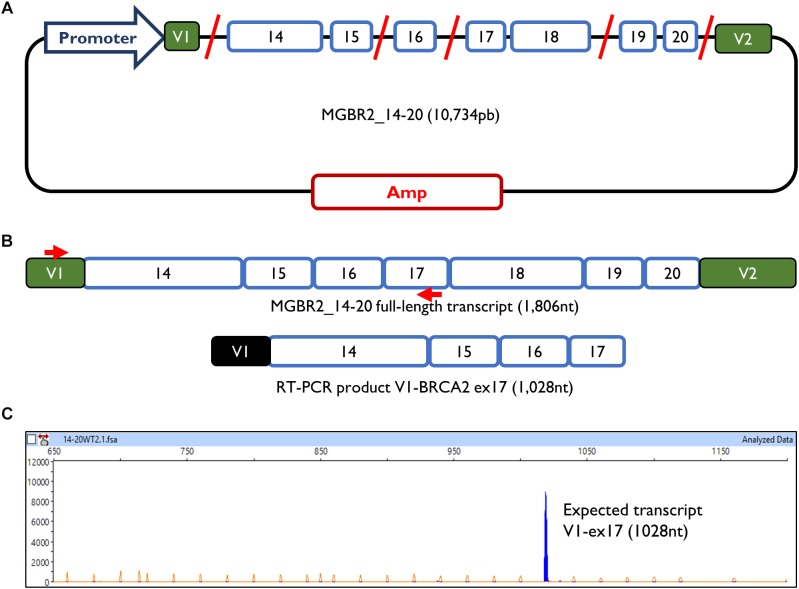
Minigene MGBR2_14-20. **(A)** Schematic representation of MGBR2_14-20. BRCA2 exons 14–20 are represented in blue boxes. Red lines represented the shortened introns. **(B)** Expected MGBR2_14-20 transcript (1,806 nt) and the amplified RT-PCR product. Specific amplification primers are shown as red arrows. **(C)** Capillary electrophoresis result of the functional assay of the wild type MGBR2_14-20 in MCF-7 cells. The full-length transcript is shown as a blue peak. The Genescan Liz-1200 size standard is shown as orange/faint peaks. Fragment sizes (nt) and relative fluorescent units are indicated on the *x*- and *y*-axes, respectively.

The minigene MGBR2_14-20 had previously been used to study variants of exons 16, 17, and 18, proving that it is a reliable and robust tool to functionally assay splicing variants (Fraile-Bethencourt et al., 2017, 2018; Montalban et al., 2018). The MGBR2_14-20 is a 10.7 Kb construct which, after transfection in MCF-7 cells, produces a transcript with the following structure: V1-BRCA2_exons from 14 to 20-V2 (1,806 nt) (Figure 1). To study exons 14 and 15, cDNA was amplified with a forward primer located in V1 (pMAD_607FW) and a reverse primer located in exon 17 (RTBR2_Ex17RV2), with an expected transcript size of 1028 nt (Figure 1B,C).

**FIGURE 2:**
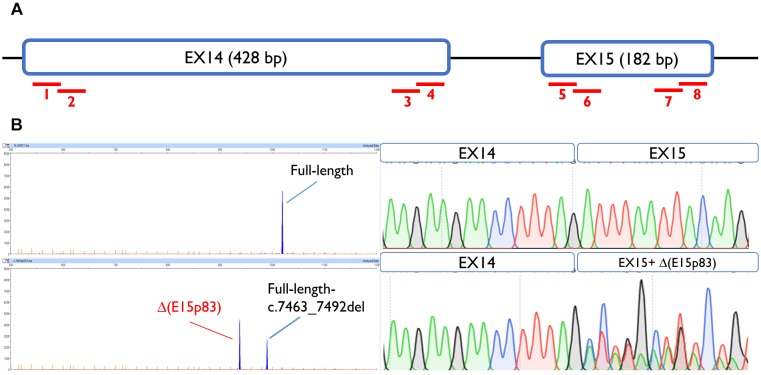
Exon 15 ESE mapping: functional assay of c.7463_7492del positive microdeletion. **(A)** Schematic representation of BRCA2 exons 14 and 15 and the eight microdeletions: 1, c.7010_7039del; 2, c.7035_7064del; 3, c.7378_7407del; 4, c.7402_7432del; 5, c.7438_7467del; 6, c.7463_7492del; 7, c.7561_7590del; 8, c.7586_7615del. **(B)** Capillary electrophoresis and sequence results of functional assays of microdeletion c.7463_7492del in MCF-7 cells.

To map ESEs, 30-nt overlapping microdeletions were performed along the first and the last 55-nt of exons 14 and 15 (Acedo et al., 2015), always preserving the splice site conserved positions (the first 2 nt and the last 3 nt of the exon). None of the deletions but one altered splicing, suggesting the absence of *cis*-regulatory motifs within these segments. Only exon 15 deletion c.7463_7492 impaired splicing generating a major aberrant transcript (62%) with a deletion of 83 nt [Δ(E15p83)]. This transcript was caused by use of a cryptic 3′ss 83-nt downstream, which is stronger than the canonical one, according to Max Ent Scan (MES; 6.18 vs. 5.16) (Figure 2). To determine which ESEs were implied in exon 15 recognition, siRNA experiments against the main splicing factors (SFSR1, SFSR2, SFSR3, SFSR5, SRSF7, SFSR9, and Tra2β) were accomplished (Supplementary Figure S1). However, none of them affected the recognition of exons 14 and 15.

### Variant Collection and Bioinformatics Analysis

A total of 294 different variants, spread throughout exons 14 and 15 and flanking introns, were collected from the main databases (ClinVar, UMD and BIC) (Supplementary Table S1). They were analyzed *in silico* with MES and NNSplice for splice site prediction, and with ESE/ESS estimation algorithms integrated in Human Splicing Finder (HSF). Potential splicing variants were selected following these criteria: creation or disruption of splice sites (according to MES or NNSplice); disruption of the branch point; disruption of the polypyrimidine tract; elimination of enhancers or creation of silencers. Some of the selected variants had a combined effect, for example, they were predicted to simultaneously create an ESS and removed an ESE. A total of 53 candidate variants (∼18%) that included 19 intronic, 18 missense, 5 nonsense, 8 synonymous, and 3 frameshift variants were selected (Table 1). According to their previous clinical classification, the selection contained: 8 benign or likely benign variants, 30 VUS and 15 pathogenic or likely pathogenic variants.

**Table 1:** Bioinformatics analysis of *BRCA2* exons 14 and 15 selected variants.

HGVS variants	Splice site		ESEs/ISEs^1^	ESS	Summary of predictions^2^
	*NNSplice*	*MaxEnt*	*ESE Finder + other motifs*	*hnRNPA1 by HSF*	
*Exon 14 (c.7008_7435)*	3′SS: 0.56 5′SS: < 0.4	3′SS: 10.37 5′SS: 5.64			
c.7008-5T > C	3′SS, 0.56→0.53	3′SS, 10.37→8.48			↓ 3′SS
c.7008-3C > G	3′SS, 0.56→ < 0.4	3′SS, 10.37→1.64			[−] 3′SS
c.7008-2A > T	3′SS, 0.56→ < 0.4	3′SS, 10.37→2.0			[−] 3′SS
c.7008-1G > A	3′SS, 0.56→ < 0.4	3′SS, 10.37→1.62			[−] 3′SS
c.7009A > G			[+] 3 ESEs; [−] 1 ESE	[+] 67.62	[−]ESEs; [+] ESS
c.7010C > T			[+]1 ESE; [−]1 ESE		[−]ESEs
c.7024C > T			[−] 2 ESEs	[+] 71.67	[−]ESEs; [+] ESS
c.7030A > G	5′SS, <0.4→0.71	5′SS, −1.55→6.62	[+]2 ESEs	[+] 76.90	[+] 5′SS; [−]ESEs; [+] ESS
c.7037A > G			[−] 2 ESEs	[+] 72.38	[−]ESEs; [+] ESS
c.7157C > A			[−] 2 ESEs	[+] 69.76	[−]ESEs; [+] ESS
c.7170T > G			[+]2 ESEs	[+]2 (67.62;76.43)	[+]ESEs; [+] ESS
c.7177A > G			[−] 1 ESE	[+] (69.52)	[−]ESEs; [+] ESS
c.7180A > T	3′SS, <0.4→0.49		[+]1 ESE	[−] (67.14)	[+] 3′SS
c.7182A > G	3′SS, <0.4→0.45				[+] 3′SS
c.7203A > G	3′SS, <0.4→0.46		[−] 2 ESEs	[+] (72.86)	[+] 3′SS; [−]ESEs; [+] ESS
c.7261C > G			[−] 1 ESE	[+] (72.38)	[−]ESEs; [+] ESS
c.7266T > A	5′SS, <0.4→0.76	5′SS, 2.7→8.34			[+] 5′SS
c.7294A > G			[−] 2 ESEs	[+] (65.48)	[−]ESEs; [+] ESS
c.7296A > G			[−] 4 ESEs	[+] (74.76)	[−]ESEs; [+] ESS
c.7330G > T			[−] 2 ESEs	[+] (68.33)	[−]ESEs; [+] ESS
c.7339A > G				[+] (71.43)	[+] ESS
c.7397C > T			[−] 4 ESEs	[+] (70.24)	[−]ESEs; [+] ESS
c.7418G > A	5′SS, <0.4→0.58		[+]2 ESEs		[+] 5′SS
c.7428A > G				[+] (71.43)	[+] ESS
c.7435+1G > C		5′SS, 5.64→-2.62			[−] 5′SS
c.7435+3A > G		5′SS, 5.64→-1.06			[−] 5′SS
c.7435+5T > C		5′SS, 5.64→5.56			[−] 5′SS
c.7435+6G > A			[−] 1 ISE		[−]ISE; +6 conserved nt;
c.7435+7T > G			[−] 1 ISE	[−] (70.48)	[−]ISEs; [−] ESS
c.7435+10G > A			[+]1 ISE; [−]1 ESE	[−] (70.48)	[−]ISEs; [−] ESS
*Exon 15 (c.7436_7617)*	3′SS: 0.9 5′SS: 0.99	3′SS: 5.16 5′SS: 9.8			

**HGVS variants**	**Splice site**		**ESEs**	**ESS**	
	***NNSplice***	***MaxEnt***	***ESE Finder + other motifs***	***hnRNPA1***	

c.7436-22C > T			*Branch point*: 79.34→55.5	[+](73.81)	↓ Branch point
c.7436-14T > G	3′SS, 0.90→0.81	3′SS, 5.16→2.03		[+] (69.05)	↓ 3′SS
c.7436-4A > G	3′SS, 0.9→ < 0.4	3′SS, 5.16→4.54			[−] 3′SS
c.7436-4A > T	3′SS, 0.9→ < 0.4	3′SS, 5.16→4.73			[−] 3′SS
c.7436-2A > T	3′SS, 0.9→ < 0.4	3′SS, 5.16→-3.2			[−] 3′SS
c.7436-1G > A	3′SS, 0.9→ < 0.4	3′SS, 5.16→-3.58			[−] 3′SS
c.7447A > G	3′SS, <0.4→0.73		[+] 2 ESEs; [−]1 ESE	[+]2 (66.43; 71.19)	[+] 5′SS
c.7466A > G	5′SS, <0.4→0.54	5′SS, 1.53→6.64	[+] 4 ESEs	[+] (76.90)	[+] 5′SS
c.7467T > C			[+] 1 ESE; [−] 2 ESEs	[+] (67.14)	[−]ESEs; [+] ESS
c.7471C > T			[+] 1 ESE; [−] 3 ESEs		[−]ESEs
c.7471delC			[−] 3 ESEs		[−]ESEs
c.7472A > G			[−] 3 ESEs	[−] (75.48)	[−]ESEs; [−] ESS
c.7474_7475delGA			[−] 4 ESEs	[−] (75.48)	[−]ESEs; [−] ESS
c.7492A > G			[+] 1 ESE	[+] 2 (71.43; 76.43)	[+]ESEs; [+] ESS
c.7501C > T	5′SS, <0.4→0.96	5′SS, 2.44→10.19	[−] 1 ESE	[+] (65.72)	[+] 5′SS; [−]ESEs; [+] ESS
c.7544C > T			[−] 1 ESE		[+] 5’SS; [−]ESEs
c.7598C > G		5′SS, −4.0→4.27	[−] 3 ESEs		[−]ESEs
c.7601C > T			[−] 2 ESEs		[−]ESEs
c.7611_7615delTAAAC	3′SS, <0.4→0.86		[+] 2 ESEs; [−]1 ESE	[+] (66.67)	[+] 3′SS; [±]ESEs; [+] ESS
c.7617G > A	5′SS, 0.99→0.8		[−] 2 ESEs	[−] (73.10)	[↓] 5′SS
c.7617+1G > A	5′SS, 0.99→ < 0.4	5′SS, 9.8→1.62	[−] 2 ESEs	[−] (73.10)	[−] 5′SS
c.7617+1G > T	5′SS, 0.99→ < 0.4	5′SS, 9.8→1.62	[−] 2 ESEs	[−] (73.10)	[−] 5′SS
c.7617+2T > G	5′SS, 0.99→ < 0.4	5′SS, 9.8→2.15	[+] 1 ESE	[−] (66.19)	[−] 5′SS

*^1^ESE, Exonic Splicing Enhancer; ISE; Intronic Splicing Enhancer; ESS, Exonic Splicing Silencer; ISS, Intronic Splicing Silencer. Full ESE predictions are available at https://figshare.com/s/246eed89fce86af1e0a6. ^2^Summary of predictions: [−], disruption; [+], creation; ↑, score increase; ↓, score decrease.*

Bioinformatics indicated that 13 variants disrupted the natural splice sites, three decreased their scores (one disrupted the polypyrimidine tract), 11 created new splice sites, one decreased the branch point score (HSF: 79.34→55.5), three altered intronic splicing elements (ISEs and ISSs) and 22 altered exonic splicing elements (ESEs and ESSs) (Table 1). Exceptionally, variants c.7008-5T > C (ivs13-5T > C) and c.7435+5T > C (ivs14+5T > C) were also selected because, even though the bioinformatics did not show significant score changes (MES: 10.37→8.48 and 5.64→5.56, respectively), these positions are relevant for exon recognition. Thus, c.7008-5T > C is the closest position of the polypyrimidine tract to the canonical acceptor site, and c.7435+5T > C is part of the consensus 5′ss sequence and +5 changes were previously associated with disease (Sharma et al., 2014; Montalban et al., 2018).

### Functional Splicing Assays Using the Minigene MGBR2_14-20

**FIGURE 3:**
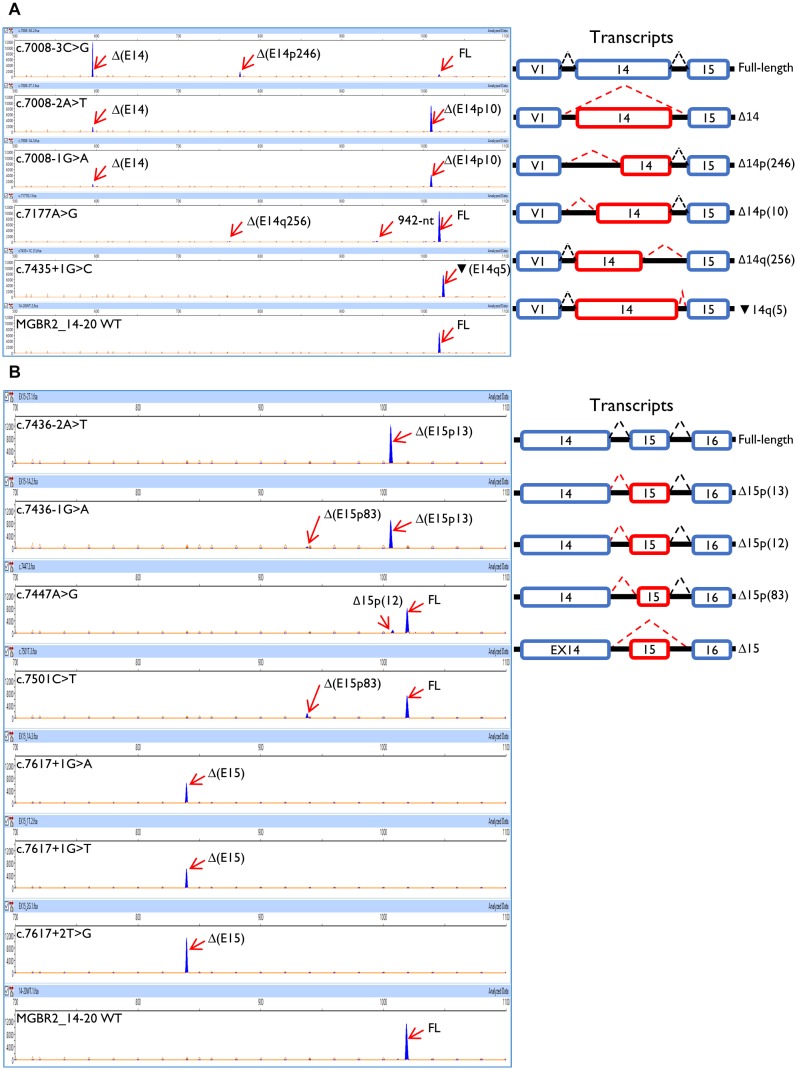
Functional assays of the BRCA2 exons 14 and 15 variants using the MGBR2_14-minigene. **(A)** Exon 14 variants. **(B)** Exon 15 variants. On the left, capillary electropherograms are shown. Transcripts were amplified using pMAD_607FW-FAM and RTBR2_Ex17RV2. Labeled transcripts are shown as blue peaks, LIZ1200 was used as size standard (orange peaks). The expected size of the full length transcript is 1028 nt (1018–1019 nt according to Peak Scanner). On the right, splicing patterns are represented. While blue boxes are natural exons, red boxes represent aberrant exons; dashed black and red lines show canonical and aberrant splicing events, respectively.

A total of 52 variants were genetically engineered in the wt MGBR2_14-20 by using specific primers (Supplementary Table S2). Despite 53 variants were initially selected, mutagenesis experiments did not work for c.7598C > G variant. The 52 mutant minigenes were checked by Sanger sequencing and assayed in MCF-7 cells. Results showed that 12 of them (23%) altered splicing (Table 2 and Figure 3), seven of which had previously been classified as pathogenic and five as VUS (Table 3). Among these 12 variants, there were 9 intronic, 2 missense and 1 nonsense changes. Functionally, the 9 intronic variants (c.7008-3C > G, c.7008-2A > T, c.7008-1G > A, c.7435+1G > C, c.7436-2A > T, c.7436-1G > A, c.7617+1G > A, c.7617+1G > T, and c.7617+2T > G) disrupted the natural splice sites, the two missense changes (c.7177A > G and c.7447A > G) triggered the use of *de novo* splice sites and originated other transcripts, and the nonsense one (c.7501C > T) probably altered SREs despite it was primarily selected because of the creation of a new 5′ss (Table 1). According to Vallée et al. (2016), nine variants induced severe splicing disruptions as they produced more than 60% of aberrant transcripts, ranging from 92.8 to 100% (Table 3).

**Table 2:** Quantification of the transcripts found by capillary electrophoresis after functional assays of *BRCA2* exons 14 and 15 variants.

Variants	Transcripts						
*Exon 14* *(c.7008_7435)*	Full-length	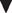 (E14q5) r.7435_7436ins 7435+1_7435+5	Δ(E14p10) r.7008_7017del	Δ(E14p246) r.7008_7253del	Δ(E14q256) r.7008_7263del	Δ(E14) r.7008_7435del	Other transcripts
c.7008-3C>G	7,2%±0.1%			13,4%±0.1%		79,3%±0.2%	
c.7008-2A>T			88.1%±0.4%			11.9%±0.4%	
c.7008-1G>A			86.5%±0.7%			13.5%±0.7%	
c.7177A>G	95.2%±0.1%				1.5%±0.02%		3.3%±0.1% (942nt)
c.7435+1G>C		100%					

*Exon 15* *(c.7436_7617)*	**Full-length**	**Δ(E15p12)** r.7436_7447del	**Δ(E15p13)** r.7436_7448del	**Δ(E15p83)** r.7436_7518del	**Δ(E15)** r.7436_7617del		

c.7436-2A>T			100%				
c.7436-1G>A			96.3%±0.1%	3.7%±0.1%			
c.7447A>G	89.9%±0.3%	10.1%±0.3%					
c.7501C>T	84.0%±0.1%			16.0%±0.1%			
c.7617+1G>A					100%		
c.7617+1G>T					100%		
c.7617+2T>G					100%		

*Short descriptions of transcripts were annotated according to the ENIGMA consortium: Δ: skipping; E: exon;*
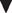
*: insertion; p: alternative 3’ss; q: alternative 5’ss; and the number of nucleotides inserted or deleted. HGVS annotations of transcripts are shown below ENIGMA description. **PTC-transcripts**:*
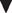
*(E14q5), Δ(E14p10), Δ(E14q256), Δ(E14), Δ(E15p13), Δ(E15p83), and Δ(E15).*

**Table 3:** Classification of spliceogenic variants.

HGVS	Type	Main RNA outcome	Protein effect	Previous classification^1^	ACMG classification^2^	ENIGMA^3^
**Exon 14 (c.7008_7435)**						
c.7008-3C > G	Intronic	r.7008_7435del	Thr2337Phefs^∗^17	VUS	PS3, PM2, PP3: L. Pathogenic	Class-4
c.7008-2A > T	Intronic	r.7008_7017del	Thr2337Asnfs^∗^27	Pathogenic	PVS1, PS3, PM2, PP3: Pathogenic	Class-4
c.7008-1G > A	Intronic	r.7008_7017del	Thr2337Asnfs^∗^27	VUS	PVS1, PS3, PM2, PP3: Pathogenic	Class-4
c.7177A > G	Missense (p.Met2393Val)	Full-length	–	VUS	PM2, PP3: VUS	Class-3
c.7435+1G > C	Intronic	r.7435_7436ins 7435+1_7435+5	Asp2479Glyfs^∗^4	Pathogenic	PVS1, PS3, PM2: Pathogenic	Class-4
**Exon 15 (c.7436_7617)**						
c.7436-2A > T	Intronic	r.7436_7448del	Asp2479Valfs^∗^41	Pathogenic	PVS1, PS3, PM2, PP3: Pathogenic	Class-4
c.7436-1G > A	Intronic	r.7436_7448del	Asp2479Valfs^∗^41	VUS	PVS1, PS3, PM2, PP3: Pathogenic	Class-4
c.7447A > G	Missense (p.Ser2483Gly)	Full-length	–	VUS	PM2, PP3: VUS	Class-3
c.7501C > T	Nonsense (p.Gln2501Ter)	Full-length	–	Pathogenic	PVS1, PM2, PP3: Pathogenic (nonsense)	Class-5 (nonsense)
c.7617+1G > A	Intronic	r.7436_7617del	Asp2479Alafs^∗^8	Pathogenic	PVS1, PS3, PM2, PP3: Pathogenic	Class-4
c.7617+1G > T	Intronic	r.7436_7617del	Asp2479Alafs^∗^8	Pathogenic	PVS1, PS3, PM2, PP3: Pathogenic	Class-4
c.7617+2T > G	Intronic	r.7436_7617del	Asp2479Alafs^∗^8	Pathogenic	PVS1, PS3, PM2, PP3: Pathogenic	Class-4

*^1^VUS, Variants of Uncertain Clinical Significance. ^2^ACMG criteria: PVS1, null variant (nonsense, frameshift, canonical ± 1 or ±2 ss, etc.) in a gene where LOF is a known mechanism of disease; PS3, well-established in vitro or in vivo functional studies supportive of a damaging effect on the gene or gene product (PS3 was only used for severe splicing alterations according to Vallée et al., 2016); PM2, absent from controls in Exome Sequencing Project, 1000 Genomes Project, or Exome Aggregation Consortium; PP3, multiple lines of computational evidence support a deleterious effect on the gene or gene product (conservation, evolutionary, splicing impact, etc.). ^3^ENIGMA criteria: Class 4: Variant considered extremely likely to alter splicing based on position, namely IVS ± 1 or IVS ± 2, or G > non-G at last base of exon if first 6 bases of the intron are not GTRRGT and/or variants are predicted bioinformatically to alter the use of the native donor/acceptor site. Class 3: “In the absence of clinical evidence to assign an alternative classification, variant allele tested for mRNA aberrations is found to produce mRNA transcript(s) predicted to encode intact full-length protein...”*

#### Acceptor Site Variants

Exon 14 3′ss was affected by c.7008-3C > G, c.7008-2A > T, and c.7008-1G > A, whereas c.7008-5T > C only produced the canonical transcript (Table 2). Curiously, while the main outcome of c.7008-3C > G was exon skipping (ΔE14), the variants c.7008-2A > T and c.7008-1G > A produced mostly the aberrant transcript Δ(E14p10), in which a cryptic 3′ss was recognized by the spliceosome 10-nt downstream. This cryptic 3′ss was not detected by NNSplice or MES tools. The loss of 10-nt at the beginning of exon 14 would generate a PTC 27 codons downstream (p.Thr2337Asnfs^∗^27). Our results also revealed the use of another cryptic 3′ss (MES = 4.44) within exon 14, located 246-nt downstream the natural one, originating the transcript Δ(E14p246) (13%) in the c.7008-3C > G assay (Table 2). The transcript Δ(E14p246) led to an in-frame deletion of 82 amino acids from position p.2337 to p.2418 (p.Thr2337_Arg2418del).

The branch point (c.7436-22C > T), polypyrimidine tract (c.7436-14T > G) and −4 (c.7436-4A > G, c.7436-4A > T) variants of exon 15 did not impair splicing. Other exon 15 acceptor variants, such as c.7436-2A > T and c.7436-1G > A, mainly caused isoform Δ(E15p13) through use of a cryptic acceptor 13-nt downstream (Table 2). The use of this cryptic acceptor would provoke a frameshift deletion, leading to a PTC in the protein (p.Asp2479Valfs^∗^41). Like exon 14 cryptic acceptor, this exon 15 cryptic 3′ss was not detected by NNSplice or MES. Variant c.7436-1G > A also produced the minor transcript ΔE15p83 (3.7%) a 83-nt deletion that introduced a frameshift (p.Asp2479Alafs^∗^32) and a PTC 32 codons downstream. This transcript was generated by a cryptic acceptor site 83-nt downstream (MES = 6.28). In summary, we found 5 variants (c.7008-3C > G, c.7008-2A > T, c.7008-1G > A, c.7436-2A > T, and c.7436-1G > A) that altered 3′ss recognition of exons 14 and 15, leading to aberrant splicing (Table 2). Remarkably, all of them showed the total absence of canonical transcript, except for c.7008-3C > G that produced 7% of the full-length transcript. Moreover, our results unveiled exon 14 and 15 cryptic splice sites that are only recognized when natural acceptors are disrupted.

#### Donor Site Variants

Seven variants were predicted to disrupt donor sites: c.7435+1G > C, c.7435+3A > G, c.7435+5T > C, and c.7435+6G > A (exon 14) and c.7617+1G > A, c.7617+1G > T, and c.7617+2T > G (exon 15; Table 1). Among the exon 14 variants, only c.7435+1G > C impaired splicing (Table 2) producing a single transcript with a 5-nt insertion [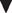
(E14q5)], due to the recognition of a cryptic 5′ss in ivs14. The 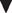
(E14q5) is an aberrant splicing isoform which leads to PTC (p.Asp2479Glyfs^∗^4). Surprisingly, this cryptic donor was not detected by NNSplice software as the canonical one was. Regarding exon 15 donor variants, our results showed that all of them (c.7617+1G > A, c.7617+1G > T, and c.7617+2T > G) produced Δ(E15) as unique transcript (Table 2), which generates a PTC eight codons downstream (p.Asp2479Alafs^∗^8).

#### Splicing Regulatory Element-Variants

A total of 26 SRE variants were selected according to the criteria above described and assayed in MCF-7 cells (Table 1). Only c.7177A > G altered weakly splicing resulting in about 5% of aberrant transcripts (Table 2). This matches with the creation of a new donor site that was not detected by the splicing prediction software. Conversely, none of the exon 15 SRE-variants impaired splicing even though microdeletion tests had revealed a presumed ESE interval (c.7463_7492).

#### New Splice Site Variants

We have assayed 10 variants of this type, six in exon 14 (c.7030A > G, c.7180A > T, c.7182A > G, c.7203A > G, c.7266T > A, and c.7418G > A) and four in exon 15 (c.7447A > G, c.7466A > G, c.7501C > T, and c.7611_7615delTAAAC) (Table 1). Results showed that two exon 15 variants (c.7447A > G and c.7501C > T) slightly disrupted splicing (Table 2). The variant c.7447A > G generated a new acceptor but most of its outcome was a full-length transcript (Table 2). The variant c.7501C > T was predicted to create a new strong donor (NNSplice: < 0.4→0.96 and MES: 2.44→10.19) (Table 1). However, only 16% of the transcripts made use of this cryptic donor 83-nt upstream of the natural one (Table 2).

### Analysis of Transcripts

The so called full-length or canonical transcript (expected size: 1028-nt) was amplified with primers placed on vector exon V1 and BRCA2 exon 17. Apart from the canonical transcript, we have detected other ten different ones (Figure 3). Aberrant exon 14 splicing produced six different isoforms, but only five of them were characterized: Δ(E14), Δ(E14p10), Δ(E14p246), Δ(E14q256), and 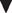
(E14q5). A 942-nt transcript of unknown structure could also be detected by capillary electrophoresis. All exon 14 isoforms, except for Δ(E14p246), introduced PTCs into the mRNA. The isoform Δ(E14p246) was seen in up to 13% of the c.7008-3C > G transcripts (Table 2). Exon 15 variants produced 4 different isoforms besides the canonical one: Δ(E15p12), Δ(E15p13), Δ(E15q83), and Δ(E15). The Δ(E15p13), Δ(E15q83) and Δ(E15) isoforms created PTCs while Δ(E15p12) (new acceptor 12-nt downstream) kept the reading frame, although this isoform only accounted for 10% of the transcripts (Table 2).

## Discussion

Due to the implementation of Next Generation Sequencing in the clinical setting (Slavin et al., 2015), a large number of variants have been detected in disease-responsible genes. HBOC and the breast cancer susceptibility genes are not exceptions, where thousands of different variants have been reported although many of them are considered as VUS (Spurdle et al., 2012; Slavin et al., 2017). In this context, the functional and clinical classifications pose a challenge for Medical Genetics. We have herein functionally assayed 52 *BRCA2* variants using the minigene MGBR2_14-20, whose reliability had been previously proven (Fraile-Bethencourt et al., 2017, 2018; Montalban et al., 2018). We found 12 variants that altered splicing, nine of which would severely alter the protein. This study forms part of a comprehensive study of our group concerning potential splicing *BRCA2* variants, where 22 exons and up to 335 variants have been assayed using three minigenes: MGBR2_2-9 (Fraile-Bethencourt et al., 2019), MGBR2_14-20 (Fraile-Bethencourt et al., 2017, 2018) and MGBR2_19-27 (Acedo et al., 2012, 2015). The following advantages of the minigene technology should be underlined: (i) analysis of a single allele outcome without the interference of the wt counterpart of a patient sample; (ii) simple and fast quantification of generated transcripts by fluorescent capillary electrophoresis with minimum hands-on time versus other proposed methods (Farber-Katz et al., 2018); (iii) versatility, one single multi-exon minigene allows to assay variants from different exons; (iv) capability of analysis in many cell types to check effects derived from tissue-specific alternative splicing; (v) high reproducibility of splicing physiological/pathological patterns. In fact, we have previously provided many examples of the minigene reproducibility. In the case of *BRCA2* exons 14 and 15, variants c.7008-2A > T, c.7617+1G > A, and c.7617+2T > G displayed similar patterns in patient RNA and minigene assays (Vreeswijk et al., 2009; Colombo et al., 2013; de Garibay et al., 2014). Moreover, another 31 variants of this and other constructs replicated previously reported patient splicing outcomes (Acedo et al., 2015; Fraile-Bethencourt et al., 2017, 2018, 2019), confirming that splicing reporters are robust and valuable tools to test the impact of variants on splicing, especially when patient RNA is not available.

**Table 4:** Summary of spliceogenic variants tested in minigene MGBR2_14-20.

DNA variant	Splicing motif^1^	Splicing outcome^2^	Clinical interpretation
**Exon 14 (this work)**			
c.7008-3C > G	[−] 3′SS	Δ(E14) 79,3%; Δ(E14p246) 13,4%; CT 7.2%	Likely Pathogenic
c.7008-2A > T	[−] 3′SS	Δ(E14p10) 88.1%; Δ(E14) 11.9%	Pathogenic
c.7008-1G > A	[−] 3′SS	Δ (E14p10) 86.5%; Δ (E14) 13.5%	Pathogenic
c.7177A > G	[−]ESE/[+]ESS	Δ (E14q256) 1.5%; 942-nt, 3.3%	VUS
c.7435+1G > C	[−] 5′SS	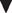 (E14q5) 100%	Pathogenic
**Exon 15 (this work)**			
c.7436-2A > T	[−] 3′SS	Δ (E15p13) 100%	Pathogenic
c.7436-1G > A	[−] 3′SS	Δ (E15p13) 96.3%; Δ (E15p83) 3.7%	Pathogenic
c.7447A > G	[+] 3′SS	Δ (E15p12) 10.1%; CT 89.9%	VUS
c.7501C > T	[−]ESE/[+]ESS	Δ (E15p83) 16%; CT 84%	Pathogenic (Nonsense)
c.7617+1G > A	[−] 5′SS	Δ (E15) 100%	Pathogenic
c.7617+1G > T	[−] 5′SS	Δ (E15) 100%	Pathogenic
c.7617+2T > G	[−] 5′SS	Δ (E15) 100%	Pathogenic
**Exon 16 (Fraile-Bethencourt et al., 2018)**			
c.7618-2A > T	[−] 3′SS	Δ (E16p44) 96.9%; Δ (E16p55) 1.8%; Δ (E16) 1.3%	Pathogenic
c.7618-2A > G	[−] 3′SS	Δ (E16p44) 97.2%; Other transcripts 2.8%	Pathogenic
c.7618-1G > A	[−] 3′SS	Δ (E16p44) 91.5%; Δ (E16p55) 4.7%; Others 2.4%	Pathogenic
c.7618-1G > C	[−] 3′SS	Δ (E16p44) 92.6%; Δ (E16) 2.8%; Δ (E16p55) 1.9%	Pathogenic
c.7802A > G	[+] 5’SS	Δ (E16q4) (45.7%); CT 54.3%	Pathogenic
c.7805G > C	[−] 5’SS	Δ (E16) 77.6%; Δ (E16q100) 14.4%; 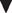 (E16q20) 6.5%	Pathogenic
c.7805+1G > A	[−] 5′SS	Δ (E16) 88%; Δ (E16q100) 10.1%	Pathogenic
c.7805+3A > C	[−] 5′SS	Δ (E16) 75.3%; Δ (E16q100) 13.3% 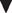 (E16q20) 3.8%	Pathogenic
**Exon 17 (Fraile-Bethencourt et al., 2017; Montalban et al., 2018)**			
c.7806-9T > G	Pyr	Δ (E17) 41.5%; 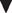 (E17p8) 36.3%; Δ (E17p69) 22.2%	Likely Pathogenic
c.7806-2A > G	[−] 3′SS	Δ (E17p20) 51.8%; Δ (E17p69) 28.1%; Δ (E17) 20.1%	Pathogenic
c.7806-1G > A	[−] 3′SS	Δ (E17p1) 100%	Pathogenic
c.7806-1G > T	[−] 3′SS	Δ (E17p20) 100%	Pathogenic
c.7806-1_7806-2dup	[+]3′SS	Δ (E17p2) 92.6%; Δ (E17) 5.1%; Δ (E17p69) 2.3%	Pathogenic
c.7975A > G	[−] 5′SS	Δ (E17) 26.2%; CT 73.8%	VUS
c.7976G > C	[−] 5′SS	Δ (E17) 100%	Likely Pathogenic
c.7976G > A	[−] 5′SS	Δ (E17) 100%	Likely Pathogenic
c.7976+1G > A	[−] 5′SS	Δ (E17) 100%	Pathogenic
c.7976+5G > T	[−] 5′SS	Δ (E17) 100%	Likely Pathogenic
**Exon 18 (Fraile-Bethencourt et al., 2017)**			
c.7977-7C > G	[+]3′SS/Pyr	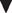 (E18p6) 78.4%; Δ (E18) 21.6%	Likely Pathogenic
c.7977-6T > G	Pyr	CT 66.7%; Δ (E18) 31%; Δ (E18p191) 2.3%	VUS
c.7977-3_7978del	[−] 3′SS	Δ (E18) 90%; Δ (E18p191) 10%	Pathogenic
c.7977-2A > T	[−] 3′SS	Δ (E18) 93.3%; Δ (E18p191) 6.7%	Pathogenic
c.7977-1G > T	[−] 3′SS	Δ (E18) 91.5%; Δ (E18p191) 7%; Δ (E18p236) 1.5%	Pathogenic
c.7977-1G > C	[−] 3′SS	Δ (E18) 89.8%); Δ (E18p191) 10.2%	Pathogenic
c.7985C > G	[−]ESE/[+]ESS	Δ (E18) 90.2%; Δ (E18p191) 5%; others 4.8%	Likely Pathogenic
c.7988A > T	[+]5′SS [−]ESE	CT 84.2%; Δ (E18) 8.6%; others 7.2%	VUS
c.7992T > A	[−]ESE/[+]ESS	CT 68.6%; Δ (E18) 31.4 %	VUS
c.8007A > G	[−]ESE/[+]ESS	CT 84.8%; Δ (E18) 15.2%	VUS
c.8009C > A	[−]ESE/[+]ESS	Δ (E18) 91.2%; Δ (E18p191) 4.8%; CT 4%	Pathogenic
c.8009C > T	[−]ESE/[+]ESS	CT 76.6%; Δ (E18) 23.4%	VUS
c.8009C > G	[−]ESE/[+]ESS	CT 79.9%; Δ (E18) 20.1%	VUS
c.8023A > G	[+] 5′SS	Δ (E18q309) 93%; other aberrant transcripts 7%	Likely Pathogenic
c.8035G > T	[+] 5′SS	Δ (E18q298) 93.6%; 878-nt transcript 4%; CT: 2.4%	Likely Pathogenic
c.8072C > T	[−]ESE/[ ± ]ESS	CT 94.9%; Δ (E18) 5.1%	VUS
c.8168A > G	[+]5′SS	CT 69.6%; Δ (E18q164) 25.9%; Δ (E18) 4.5%	VUS
c.8249_8250del	[−]ESE/[−]ESS	CT 93.0%; Δ (E18) 7.0%	VUS
c.8331G > A	[−] 5′SS	Δ (E18) 52%; CT 40.7%; aberrant transcripts 7.3%	Likely Pathogenic
c.8331+1G > T	[−] 5′SS	Δ (E18) 81%; Ex18-del157 6.4%; Δ (E17q151, E18) 6.1%; 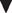 (E17q58)+ Δ (E18) 3.7%; others 2.8%	Pathogenic
c.8331+2T > C	[−] 5′SS	Δ (E18) 87.1%; Δ (E17q151,E18) 12.9%	Pathogenic

*^1^[−], disruption; [+], creation; [±], simultaneous creation and disruption; 5′SS, 5′ splice site; 3′SS, 3′ splice site; ESE, Exonic Splicing Enhancer; ESS, Exonic Splicing Silencer; Pyr, polypyrimidine tract. ^2^Transcript annotation according to Lopez-Perolio et al. (2019); CT, canonical transcript.*

Here, we have shown that nine variants drastically disrupted the splicing pattern. We found five 3′ss disrupting variants, one of which (c.7008-3C > G) provoked exon 14 skipping, whereas the rest of them induced the use of cryptic sites (c.7008-2A > T, c.7008-1G > A, c.7436-2A > T, and c.7436-1G > A). Moreover, variant c.7435+1G > C, which disrupt the exon 14 donor site, provoked the use of a cryptic donor. Curiously, neither c.7435+3A > G, c.7435+5T > C nor c.7435+6G > A, which are also part of the consensus 5′ss, affected splicing. This may be due to the low frequency of +5T and +6G at these positions, so that any nucleotide change only equals or improves splice site strength. Conversely, +3A is the main nucleotide at this position (71%) but +3G is also relatively frequent (24%) (Zhang, 1998). Thus, a substitution A to G might have a reduced or no splicing impact, as it is the case of the c.7435+3A > G variant. As a matter of fact, variant c.9501+3A > T produced 87% of the canonical transcript (Acedo et al., 2015). On the other hand, the three variants of exon 15 donor site c.7617+1G > A, c.7617+1G > T, and c.7617+2T > G resulted in exon skipping. In this regard, it was recently recommended the use of a combination of the computational tools HSF plus Splice Site Finder-like to select candidate splice site variants with high sensitivity and specificity (Moles-Fernández et al., 2018). According to HSF, variants c.7435+3A > G, c.7435+5T > C, and c.7435+6G > A showed only minimal changes of the splice site scores (±1%) so that they should have been excluded from subsequent functional tests.

Concerning other splicing motifs, minigenes also allow the identification of regulatory sequences (splicing enhancers and silencers) and splicing factors involved in the specific regulation (Baralle and Buratti, 2017). Indeed, the SRE mapping constitutes an interesting experimental approach since it identifies critical regions for exon recognition. In this context, our group previously found exonic variants that disrupted splicing through elimination of ESEs or creation of *de novo* silencers, such as *BRCA2* variants c.7985C > G (predicted missense p.Thr2662Arg) or c.8009C > A (predicted nonsense p.Ser2670^∗^) (Fraile-Bethencourt et al., 2017). The splicing assays showed how both variants elicited complete splicing aberrations (mainly exon 18 skipping). However, they were *a priori* classified as missense and nonsense variants, respectively, due to their predicted protein effect. After microdeletion mapping, we have identified a putative ESE-region in exon 15 (c.7463_7492). These ESEs might be involved in exon 15 3′ss recognition since their loss produced the use of a cryptic acceptor 83-nt downstream (Figure 2). Curiously, we did not find any ESE-variants that affected pre-mRNA processing. Only, c.7501C > T, which lays near to this presumed ESE interval, provoked a similar outcome to that of r.7463_7492 deletion (Table 2). In summary, we have tested a total of 117 different variants in minigene MGBR2_14-20, from exons 14–18 (Table 4), 51 of which (43.6%) induced abnormal splicing patterns: 31 disrupted the natural splice sites (16 3′ss and 15 5′ss), 11 affected SREs, six created *de novo* splice sites and three altered the polypyrimidine tract.

### Transcript Interpretation

The *BRCA2* exons 14 (c.7008_7435) and 15 (c.7436_7617) encode for amino acids 2336 to p.2539, which are part of the DNA Binding Domain (DBD; p.2459_p.3190). The DBD is the largest conserved region of BRCA2 and is composed of a helical domain (HD), three oligonucleotide binding sites (OB) and a tower domain (TD) (Guidugli et al., 2014). Specifically, exons 14 and 15 are part of the HD (p.2481_2667) that binds to the protein DSS1 (deleted in split-hand/split-foot syndrome) in the region comprised by the residues 2472–2957 (Marston et al., 1999). Among these residues, a total of 125 are strictly conserved from human to sea urchin. DSS1 is important for BRCA2 stability, since its loss leads to a reduction of BRCA2 levels in human cells (Li et al., 2006). Moreover, exons 14 and 15 coding region is also recognized by FANCD2 (Fanconi anemia group D2) protein, which binds to the BRCA2 protein between codons 2350 and 2545 (Hussain et al., 2004). FANCD2, like BRCA2, is one of the 16 proteins that form the Fanconi Anemia complex, aimed to repair DNA interstrand crosslinks. However, it was shown that BRCA2-FANCD2 association has an independent function in the Fanconi Anemia pathway. The BRCA2-FANCD2 complex is involved in the restart of the replication fork, by protecting the nascent DNA strands from degradation (Raghunandan et al., 2015). Taken together, exons 14 and 15 contain crucial sequences of *BRCA2*, owing to its function in homologous recombination and other relevant biological processes. Moreover, the biological relevance of exons 14 and 15 is supported by the presence of numerous pathological nonsense and frameshift variants at the mutation databases. Hence, the exon 14–15 spliceogenic variants that induce PTC-transcripts may be associated with an increased risk of HBOC.

### Clinical Interpretation of Spliceogenic Variants

Twelve variants altered splicing with different patterns. While some variants caused the total absence of canonical transcript, others originated just ∼5% of aberrant transcripts (Table 2). Variants c.7008-2A > T, c.7008-1G > A, c.7435+1G > C, c.7436-2A > T, c.7436-1G > A, c.7617+1G > A, c.7617+1G > T, and c.7617+2T > G did not produce the canonical transcript. Moreover, all the transcripts generated by these variants were frameshift transcripts. Thus, following the criteria of the American College of Medical Genetics and Genomics (ACMG), these eight variants should be classified as pathogenic variants (Table 3). Variant c.7008-3C > G produced Δ(E14) as the major transcript, but the full-length (∼7%) and the in-frame transcript Δ(E14p246) (∼13%) were also identified. The Δ(E14p246) isoform contains a deletion of 82 non-conserved amino acids (p.Thr2337_Arg2418del) that form part of the FANCD2 binding site (p.Thr2350_Val2545). At the UMD database, c.7008-3C > G is classified as a “causal” variant because of the skipping of exon 14, but ClinVar shows it as VUS. According to the ACMG criteria, this variant should be cataloged as likely pathogenic (Table 3). On the other hand, according to the guidelines of the ENIGMA consortium^7^ eight variants should be classified as Class 4 (Likely pathogenic), three as VUS and one as pathogenic, though this is due to its predicted nonsense effect (Table 3).

On the other hand, missense variants c.7177A > G (p.Met2393Val) and c.7447A > G (p.Ser2483Gly) produced the canonical transcript as the main outcome and only 5 and 10% of aberrant isoforms, respectively (Table 2). The canonical transcript generated by these two variants carried missense changes, but *in silico* predictions with the PolyPhen tool^8^ suggested that p.Met2393Val and p.Ser2483Gly were both benign changes. So, following the ACMG criteria, c.7177A > G and c.7447A > G remain as VUS (Table 3). Finally, variant c.7501C > T generated mainly the canonical transcript (84%) that includes a nonsense change (pathogenic according to ClinVar) so this change should be classified as pathogenic under a combined splicing-protein viewpoint. Altogether, we have re-classified three variants (c.7008-3C > G, c.7008-1G > A, and c.7436-1G > A) from VUS to pathogenic or likely pathogenic, and we have provided further support for the classification of six spliceogenic variants (c.7008-2A > T, c.7435+1G > C, c.7436-2A > T, c.7617+1G > A, c.7617+1G > T, and c.7617+2T > G). Interestingly, c.7617G > A is classified as “causal” in the UMD database^9^ and indicated that causes exon 15 skipping but no functional proofs were provided. However, the functional assay of MGBR2_14-20-c.7617G > A only showed the canonical transcript (Supplementary Figure S2). In fact, NNSplice, HSF and MES estimated just a small decrease (−19, −11.6, and −24%, respectively) of the donor site score. Therefore, c.7617G > A behaves as a neutral variant from the splicing perspective. Finally, the minigene MGBR2_14-20 results of 5 exons suggest that a total of 39 spliceogenic variants should be classified as pathogenic or likely pathogenic (Table 4), lending further support to this strategy for the clinical interpretation of variants.

In summary, splicing is a finely regulated mechanism which can be altered by any change in the sequence. The disruption of this process might cause serious effects on the protein, from the loss of important domains to the generation of a PTC. Thus, splicing alteration is a common mechanism of gene inactivation, which is often involved in human disease. Here, we have revealed 12 spliceogenic variants of *BRCA2* exons 14 and 15. The minigene based assays offer a relevant information about effects of splicing variants, since they allow to functionally assay almost any DNA change, to quantify all generated transcripts, including very rare ones, and to initially study the splicing regulation. Moreover, we have detected an ESE region that seems to be regulating exon 15 splicing, and therefore constitutes a hypothetical hotspot for putative ESE-mutations. Indeed, pSAD-based minigenes have constituted an invaluable technology to functionally test variants of other disease genes such as *GRN* (Frontotemporal Dementia), *SERPINA1* (Severe alpha-1 antitrypsin deficiency) and *CHD7* (Charge Syndrome) genes (Lara et al., 2014; Villate et al., 2018). Altogether, these results highlight, once more, the importance of RNA assays to know the splicing effects of DNA variants to give support to their clinical interpretation and consequently to activate preventive and/or therapeutic interventions.

## Author Contributions

EF-B contributed to the bioinformatics analysis, minigene construction, manuscript writing, and performed most of the splicing functional assays. BD-G and AV-P participated in minigene construction, mutagenesis experiments, and functional assays. SG-B and MC carried out the data collection of variants and their computer analysis, as well as manuscript editing. EV conceived the study and the experimental design, supervised all the experiments, and wrote the manuscript. All authors contributed to data interpretation, revisions of the manuscript, and approved the final version of the manuscript.

## Conflict of Interest Statement

The authors declare that the research was conducted in the absence of any commercial or financial relationships that could be construed as a potential conflict of interest.

## Footnotes

1 https://www.ncbi.nlm.nih.gov/clinvar

2 https://www.ncbi.nlm.nih.gov/clinvar/

3 http://www.umd.be/BRCA2/

4 https://research.nhgri.nih.gov/projects/bic/index.shtml

5 http://www.fruitfly.org/seq_tools/splice.html

6 http://www.umd.be/HSF3/

7 https://enigmaconsortium.org/library/general-documents/enigma-classification-criteria/

8 http://genetics.bwh.harvard.edu/pph2/

9 https://goo.gl/bG2V4n
