# Supplementary material for: Minigene Splicing Assays Identify 12 Spliceogenic Variants of BRCA2 Exons 14 and 15
Source: Front Genet. 2019 May 28;10:503. doi: 10.3389/fgene.2019.00503 (PMC6546720; doi:10.3389/fgene.2019.00503)
Supplement: Supplementary file 1 [file Data_Sheet_1.PDF]

Supplemental Table S1. Collection of 294 different variants within studied region, from ClinVar, UMD and BIC. Variants are noted following the HGVS criteria.

Variants in exon 14 and flanking intronic regions
c.7008-20_c.7008-17del4
c.7008-20A>G
c.7008-17T>G
c.7008-15delTT
c.7008-14delT
c.7008-13C>T
c.7008-10C>T
c.7008-5T>C
c.7008-3C>G
c.7008-2A>T
c.7008-1G>A
c.7009A>G
c.7010C>T
c.7011A>G
c.7012A>G
c.7015A>G
c.7017G>C
c.7017G>T
c.7021C>T
c.7022G>T
c.7022G>A
c.7024C>T
c.7030A>G
c.7034A>G
c.7037A>G
c.7039C>G
c.7040C>A
c.7042A>C
c.7045T>C
c.7047delT
c.7049C>T
c.7050C>T
c.7051G>A
c.7052C>G
c.7053A>T
c.7055C>T
c.7057G>C
c.7060C>T
c.7063G>T
c.7066T>G
c.7066T>C
c.7067T>A
c.7069C>T
c.7071G>T
c.7072T>C
c.7073C>G
c.7078T>C
c.7081C>T
c.7082A>G
c.7082A>T
c.7086G>A
c.7088A>G
c.7090G>T
c.7090G>A
c.7093C>T
c.7095T>A
c.7096C>G
c.7098G>A
c.7102T>C
c.7102T>G
c.7104G>A
c.7107A>C
c.7115C>A
c.7115C>G
c.7118G>C
c.7119C>G
c.7119C>T
c.7124T>G
c.7126G>C
c.7133C>G
c.7137A>G
c.7140T>A
c.7150C>A
c.7152A>G
c.7157C>A
c.7160C>T
c.7162A>G
c.7168A>G
c.7170T>G
c.7172A>T
c.7175A>G
c.7177A>G
c.7178T>C
c.7180A>T
c.7182A>G
c.7185C>T
c.7185C>A
c.7187T>A
c.7187T>C
c.7186_7187delTT
c.7188G>T
c.7188G>A
c.7191T>A
c.7193C>G
c.7195A>G
c.7196C>T
c.7203A>G
c.7205C>T
c.7207A>G
c.7208C>T
c.7210A>T
c.7215C>A
c.7218T>G
c.7219G>C
c.7222C>T
c.7224A>G

c.7232A>C
c.7234A>G
c.7235C>T
c.7237A>G
c.7239A>G
c.7241C>T
c.7241C>G
c.7242A>G
c.7242A>T
c.7244A>G
c.7248T>C
c.7251C>T
c.7252A>G
c.7253G>A
c.7256T>G
c.7258G>T
c.7260A>G
c.7261C>G
c.7261C>T
c.7266T>A
c.7270A>G
c.7273A>G
c.7278T>A
c.7279A>G

c.7282T>C
c.7283T>A
c.7287G>A
c.7294A>G
c.7296A>G
c.7299A>G
c.7301A>C
c.7303C>T
c.7304A>G
c.7307A>G
c.7309A>G
c.7311T>A
c.7313A>G
c.7314T>A
c.7316G>C
c.7317A>G
c.7319A>C
c.7319A>G
c.7322G>C
c.7326T>C
c.7330G>C
c.7330G>T
c.7331A>G
c.7331A>T

c.7339A>G
c.7341T>C
c.7342A>C
c.7343A>G
c.7347T>G
c.7347T>C
c.7349A>T
c.7350T>C
c.7351G>A
c.7354A>G
c.7355A>G
c.7356T>C
c.7359G>A
c.7360A>T
c.7360A>G
c.7366C>T
c.7366C>G
c.7370T>G
c.7373A>G
c.7375A>T
c.7376A>G
c.7394C>T
c.7397C>T
c.7398A>G

c.7402G>A
c.7404A>C
c.7409T>C
c.7410C>G
c.7411A>G
c.7413A>G
c.7414A>G
c.7415A>C
c.7416G>T
c.7417T>C
c.7418G>A
c.7428A>G
c.7429C>A
c.7429C>T
c.7431T>C
c.7435+1G>C
c.7435+3A>G
c.7435+5T>C
c.7435+6G>A
c.7435+7T>G
c.7435+10G>A

Variants in exon 15 and flanking intronic regions
c.7436-43T>G
c.7436-22c>T
c.7436-17T>G
c.7436-14T>G
c.7436-10T>C
c.7436-4A>G
c.7436-4A>T
c.7436-2A>G
c.7436-2A>T
c.7436-2delAGAT
c.7436-1G>A
c.7438T>G
c.7443_7443delT
c.7447A>G
c.7448G>A
c.7463G>A
c.7464A>C
c.7466A>G
c.7467_7468insT
c.7467T>C
c.7469T>C
c.7471C>T
c.7471delC
c.7472A>G
c.7474_7475delGA
c.7478T>G
c.7480C>T
c.7481G>A
c.7484T>C
c.7485dup
c.7487A>C
c.7491_7493delGAA
c.7491G>A
c.7492A>G
c.7495C>T
c.7499G>C
c.7501C>T
c.7503A>T
c.7504C>T
c.7505G>A
c.7505G>C
c.7506C>G
c.7506C>T
c.7507G>A
c.7509C>G
c.7512T>G
c.7521A>T
c.7522G>A
c.7523G>T
c.7525_7526insA
c.7527T>C
c.7528C>T
c.7529T>C
c.7534C>T
c.7537G>A
c.7542_7549dup
c.7543_7543delA
c.7543_7544insA
c.7544C>T
c.7552C>G
c.7556_7557insC
c.7558C>T
c.7559G>A
c.7559G>T
c.7561A>G
c.7561delA
c.7562T>C
c.7565_7568delCTCT
c.7565C>T
c.7567_7568delCT
c.7575A>G
c.7579delG
c.7580_7582delTAG
c.7583G>A
c.7586G>A
c.7593_7593delT
c.7595_7596insTT
c.7596_7609del
c.7596C>T
c.7597T>G
c.7598C>G
c.7600G>A
c.7601C>T
c.7602G>C
c.7610A>G
c.7611_7615delTAAAC
c.7612A>T
c.7617G>A
c.7617+1G>A
c.7617+1G>T
c.7617+2T>G
c.7617+4T>C
c.7617+14delTA
c.7617+17A>G
c.7617+19T>G

Supplemental Table S2. Mutagenesis primer sequences of 53 variants and 8 microdeletions in *BRCA2* exons 14 and 15.

HGVS	MD-Primer sequences
Exon 14 (c.7008_7435)	
**c.7008-5T>C**	5' CTTATATATTTTCTCCCCATCGCAGCACAACTAAGGAACGT 3'
	5' ACGTTCCTTAGTTGTGCTGCGATGGGGAGAAAATATATAAG 3'
**c.7008-3C>G**	5' TATATATTTTCTCCCCATTGGAGCACAACTAAGGAACGTCA 3'
	5' TGACGTTCCTTAGTTGTGCTCCAATGGGGAGAAAATATATA 3'
**c.7008-2A>T**	5' ATATATTTTCTCCCCATTGCTGCACAACTAAGGAACGTCAA 3'
	5' TTGACGTTCCTTAGTTGTGCAGCAATGGGGAGAAAATATAT 3'
**c.7008-1G>A**	5' TATATTTTCTCCCCATTGCAACACAACTAAGGAACGTCAAG 3'
	5' CTTGACGTTCCTTAGTTGTGTTGCAATGGGGAGAAAATATA 3'
**c.7009A>G**	5' TATTTTCTCCCCATTGCAGCGCAACTAAGGAACGTCAAGAG 3'
	5' CTCTTGACGTTCCTTAGTTGCGCTGCAATGGGGAGAAAATA 3'
**c.7010C>T**	5' ATTTTCTCCCCATTGCAGCATAACTAAGGAACGTCAAGAGA 3'
	5' TCTCTTGACGTTCCTTAGTTATGCTGCAATGGGGAGAAAAT 3'
**c.7030A>G**	5' CAACTAAGGAACGTCAAGAGGTACAGAATCCAAATTTTAC 3'
	5' GTAAAATTTGGATTCTGTACCTCTTGACGTTCCTTAGTTG 3'
**c.7170T>G**	5' TCTGCTACAAGAAAGGAAAAAATGAGACACTTGAT 3'
	5' ATCAAGTGTCTCATTTTTTCCTTTCTTGTAGCAGA 3'
**c.7182A>G**	5' ACAAGAAATGAAAAAATGAGGCACTTGATTACTACAGGCAG 3'
	5' CTGCCTGTAGTAATCAAGTGCCTCATTTTTTCATTTCTTGT 3'
**c.7266T>A**	5' TTTCACAGAGTTGAACAGTGAGTTAGGAATATTAACTTGG 3'
	5' CCAAGTTAATATTCCTAACTCACTGTTCAACTCTGTGAAA 3'
**c.7418G>A**	5' GCAGCTGTAACTTTCACAAAGTATGAAGAAGAACCTTTAGGTATT 3'
	5' AATACCTAAAGGTTCTTCTTCATACTTTGTGAAAGTTACAGCTGC 3'
**c.7024C>T**	5' CAGCACAACTAAGGAACGTTAAGAGATACAGAATCCAAAT 3'
	5' ATTTGGATTCTGTATCTCTTAACGTTCCTTAGTTGTGCTG 3'
**c.7037A>G**	5' GAACGTCAAGAGATACAGAGTCCAAATTTTACCGCACC 3'
	5' GGTGCGGTAAAATTTGGACTCTGTATCTCTTGACGTTC 3'
**c.7157C>A**	5' ACATCCATTTTATCAAGTTTATGCTACAAGAAAT 3'
	5' ATTTCTTGTAGCATAAACTTGATAAAATGGATGT 3'
**c.7177A>G**	5' TCAAGTTTCTGCTACAAGAAATGAAAAAGTGAGACACTTGATTAC 3'
	5' GTAATCAAGTGTCTCACTTTTTCATTTCTTGTAGCAGAAACTTGA 3'
**c.7180A>T**	5' CTACAAGAAATGAAAAAATGTGACACTTGATTACTACAGG 3'
	5' CCTGTAGTAATCAAGTGTCACATTTTTTCATTTCTTGTAG 3'
**c.7203A>G**	5' CACTTGATTACTACAGGCAGGCCAACCAAAGTCTTTG 3'
	5' CAAAGACTTTGGTTGGCCTGCCTGTAGTAATCAAGTG 3'
**c.7261C>G**	5' ACATTTTCACAGAGTTGAAGAGTGTGTTAGGAATATTAAC 3'
	5' GTTAATATTCCTAACACACTCTTCAACTCTGTGAAAATGT 3'
**c.7294A>G**	5' ATATTAACTTGGAGGAAAACGGACAAAAGCAAAACATTGAT 3'
	5' ATCAATGTTTTGCTTTTGTCCGTTTTCCTCCAAGTTAATAT 3'
**c.7296A>G**	5' ATTAACTTGGAGGAAAACAGGCAAAAGCAAAACATTGATGG 3'
	5' CCATCAATGTTTTGCTTTTGCCTGTTTTCCTCCAAGTTAAT 3'
**c.7330G>T**	5' TTGATGGACATGGCTCTGATTATAGTAAAAATAAGATTAAT 3'
	5' ATTAATCTTATTTTTACTATAATCAGAGCCATGTCCATCAA 3'
**c.7339A>G**	5' GGCTCTGATGATAGTAAAGATAAGATTAATGACAATGAG 3'
	5' CTCATTGTCATTAATCTTATCTTTACTATCATCAGAGCC 3'
**c.7397C>T**	5' AAACAACTCCAATCAAGCAGTAGCTGTAACTTTCACAAA 3'
	5' TTTGTGAAAGTTACAGCTACTGCTTGATTGGAGTTGTTT 3'
**c.7428A>G**	5' ACAAAGTGTGAAGAAGAGCCTTTAGGTATTGTATGA 3'
	5' TCATACAATACCTAAAGGCTCTTCTTCACACTTTGT 3'
**c.7435+1G>C**	5' CAAAGTGTGAAGAAGAACCTTTAGCTATTGTATGACA 3'
	5' TGTCATACAATAGCTAAAGGTTCTTCTTCACACTTTG 3'

**c.7435+3A>G**	5' TGTGAAGAAGAACCTTTAGGTGTTGTATGACAATTTGTGTGATGA 3'
	5' TCATCACACAAATTGTCATACAACACCTAAAGGTTCTTCTTCACA 3'
**c.7435+5T>C**	5' CCTTTAGGTATCGTATGACAATTTGTGTGATGAATTTTTGCC 3'
	5' GGCAAAAATTCATCACACAAATTGTCATACGATACCTAAAGG 3'
**c.7435+6G>A**	5' GAAGAAGAACCTTTAGGTATTATATGACAATTTGTGTGATGA 3'
	5' TCATCACACAAATTGTCATATAATACCTAAAGGTTCTTCTTC 3'
**c.7435+7T>G**	5' GAAGAACCTTTAGGTATTGGATGACAATTTGTGTGATGA 3'
	5' TCATCACACAAATTGTCATCCAATACCTAAAGGTTCTTC 3'
**c.7435+10G>A**	5' GAACCTTTAGGTATTGTATAACAATTTGTGTGATGA 3'
	5' TCATCACACAAATTGTTATACAATACCTAAAGGTTC 3'
**EX14-Microdeletions**	
**c.7010_7039del**	5'TATATATTTTCTCCCCATTGCAGCACAAATTTTACCGCACCTGGTCAAGA3'
	5'TCTTGACCAGGTGCGGTAAAATTTGTGCTGCAATGGGGAGAAAATATATA3'
**c.7035_7064del**	5' AACTAAGGAACGTCAAGAGATACAATTTCTGTCTAAATCTCATTTGTATG 3'
	5' CATACAAATGAGATTTAGACAGAAATTGTATCTCTTGACGTTCCTTAGTT 3'
**c.7378_7407del**	5' AATGACAATGAGATTCATCAGTTTAACAAATTCACAAAGTGTGAAGAAGAACCTTTAGGT 3’
	5' ACCTAAAGGTTCTTCTTCACACTTTGTGAATTTGTTAAACTGATGAATCTCATTGTCATT 3'
**c.7402_7432del**	5' AACAAAAACAACTCCAATCAAGCAGCAGCTTAGGTATTGTATGACAATTTGTGTGATGAA 3'
	5' TTCATCACACAAATTGTCATACAATACCTAAGCTGCTGCTTGATTGGAGTTGTTTTTGTT 3'
Exon 15 (c.7436_7617)	
**c.7436-22C>T**	5' AATTTCAATTTTATTTTTGTTAAGTATTTATTCTTTGATA 3'
	5' TATCAAAGAATAAATACTTAACAAAAATAAAATTGAAATT 3'
**c.7436-14T>G**	5' ATTTTATTTTTGCTAAGTATGTATTCTTTGATAGATTTAA 3'
	5' TTAAATCTATCAAAGAATACATACTTAGCAAAAATAAAAT 3'
**c.7436-4A>G**	5' GCTAAGTATTTATTCTTTGGTAGATTTAATTACAAGTCTT 3'
	5' AAGACTTGTAATTAAATCTACCAAAGAATAAATACTTAGC 3'
**c.7436-4A>T**	5' GCTAAGTATTTATTCTTTGTTAGATTTAATTACAAGTCTT 3'
	5' AAGACTTGTAATTAAATCTAACAAAGAATAAATACTTAGC 3'
**c.7436-2A>T**	5' CTAAGTATTTATTCTTTGATTGATTTAATTACAAGTCTTC 3'
	5' GAAGACTTGTAATTAAATCAATCAAAGAATAAATACTTAG 3'
**c.7436-1G>A**	5' TTATTCTTTGATAAATTTAATTACAAGTCTTCAGAATGC 3'
	5' GCATTCTGAAGACTTGTAATTAAATTTATCAAAGAATAA 3'
**c.7447A>G**	5' CTTTGATAGATTTAATTACAGGTCTTCAGAATGCCAGAGA 3'
	5' TCTCTGGCATTCTGAAGACCTGTAATTAAATCTATCAAAG 3'
**c.7466A>G**	5' AGTCTTCAGAATGCCAGAGGTATACAGGATATGCGAATTA 3'
	5' TAATTCGCATATCCTGTATACCTCTGGCATTCTGAAGACT 3'
**c.7467T>C**	5' TCTTCAGAATGCCAGAGACATACAGGATATGCGAATTAA 3'
	5' TTAATTCGCATATCCTGTATGTCTCTGGCATTCTGAAGA 3'
**c.7471C>T**	5' AGAATGCCAGAGATATATAGGATATGCGAATTAAGAAG 3'
	5' CTTCTTAATTCGCATATCCTATATATCTCTGGCATTCT 3'
**c.7471delC**	5' AGAATGCCAGAGATATAAGGATATGCGAATTAAGAAG 3'
	5' CTTCTTAATTCGCATATCCTTATATCTCTGGCATTCT 3'
**c.7472A>G**	5’ TCAGAATGCCAGAGATATACGGGATATGCGAATTAAGAAG 3’
	5’ CTTCTTAATTCGCATATCCCGTATATCTCTGGCATTCTGA 3’
**c.7474_7475del**	5' AGAATGCCAGAGATATACAGTATGCGAATTAAGAAGAAAC 3'
	5' GTTTCTTCTTAATTCGCATACTGTATATCTCTGGCATTCT 3'
**c.7492A>G**	5' AGGATATGCGAATTAAGAAGGAACAAAGGCAACGCGTCTT 3'
	5' AAGACGCGTTGCCTTTGTTCCTTCTTAATTCGCATATCCT 3'
**c.7501C>T**	5' GAATTAAGAAGAAACAAAGGTAACGCGTCTTTCCACA 3'
	5' TGTGGAAAGACGCGTTACCTTTGTTTCTTCTTAATTC 3'
**c.7544C>T**	5' AGTCTGTATCTTGCAAAAATATCCACTCTGCCTCGAATC 3'
	5' GATTCGAGGCAGAGTGGATATTTTTGCAAGATACAGACT 3'
**c.7598C>G**	5' CAGTAGGAGGCCAAGTTCCCTGTGCGTGTTCTCATAAAC 3'
	5' GTTTATGAGAACACGCACAGGGAACTTGGCCTCCTACTG 3'
**c.7601C>T**	5' GGAGGCCAAGTTCCCTCTGTGTGTTCTCATAAACAGGTA 3'
	5' TACCTGTTTATGAGAACACACAGAGGGAACTTGGCCTCC 3'
	5' GTTCCCTCTGCGTGTTCTCAAGGTATGTGTTTGTCTAC 3'

**c.7611_7615del**	5' GTAGACAAACACATACCTTGAGAACACGCAGAGGGAAC 3'
**c.7617G>A**	5' TCTGCGTGTTCTCATAAACAAGTATGTGTTTGTCTACAA 3'
	5' TTGTAGACAAACACATACTTGTTTATGAGAACACGCAGA 3'
**c.7617+1G>A**	5' CTGCGTGTTCTCATAAACAGATATGTGTTTGTCTACAATA 3'
	5' TATTGTAGACAAACACATATCTGTTTATGAGAACACGCAG 3'
**c.7617+1G>T**	5' CTGCGTGTTCTCATAAACAGTTATGTGTTTGTCTACAATA 3'
	5' TATTGTAGACAAACACATAACTGTTTATGAGAACACGCAG 3'
**c.7617+2T>G**	5' TTCTCATAAACAGGGATGTGTTTGTCTACAATACTGATGG 3'
	5' CCATCAGTATTGTAGACAAACACATCCCTGTTTATGAGAA 3'
**EX15-Microdeletions**	
**c.7438_7467del**	5' GCTAAGTATTTATTCTTTGATAGATATACAGGATATGCGAATTAAGAAGA 3'
	5' TCTTCTTAATTCGCATATCCTGTATATCTATCAAAGAATAAATACTTAGC 3'
**c.7463_7492del**	5' TAGATTTAATTACAAGTCTTCAGAATGCCAAACAAAGGCAACGCGTCTTTCCACAGCCAG 3'
	5' CTGGCTGTGGAAAGACGCGTTGCCTTTGTTTGGCATTCTGAAGACTTGTAATTAAATCTA 3'
**c.7561_7590del**	5' TGCAAAAACATCCACTCTGCCTCGAGTTCCCTCTGCGTGTTCTCATAAAC 3'
	5' GTTTATGAGAACACGCAGAGGGAACTCGAGGCAGAGTGGATGTTTTTGCA 3'
**c.7586_7615del**	5' ATCTCTCTGAAAGCAGCAGTAGGAGAGGTATGTGTTTGTCTACAATACTG 3'
	5' CAGTATTGTAGACAAACACATACCTCTCCTACTGCTGCTTTCAGAGAGAT 3'

Supplemental Table S3. siRNAs against SR proteins and Tra2β and qPCR primers sequences.

siRNA anti-SR protein	Sequence
**SRSF1 (SF2/ASF)**	5′ ACGAUUGCCGCAUCUACGU 3′
	5′ ACGUAGAUGCGGCAAUCGU 3′
**SRSF2 (SC35)**	5′ AAUCCAGGUCGCGAUCGAA 3′
	5′ UUCGAUCGCGACCUGGAUU 3′
**SRSF3 (SRp20)**	5′ GAGUGGAACUGUCGAAUGG 3′
	5′ CCAUUCGACAGUUCCACUC 3′
**SRSF5 (SRp40)**	5′ CCACCUGUAAGAACAGAAA 3′
	5′ UUUCUGUUCUUACAGGUGG 3′
**SRSF7 (9G8)**	5′ GGAGAGUUAGAAAGGGCUU 3′
	5′ AAGCCCUUUCUAACUCUCC 3′
**SRSF9 (SRp30c)**	5′ GGAAUAUGCCCUGCGUAAA 3′
	5′ UUUACGCAGGGCAUAUUCC 3′
**Tra2β**	5′ GGAGGAUACAGAUCACGUU 3′
	5′ AACGUGAUCUGUAUCCUCC 3′
**Luciferase**	5′ UAAGGCUAUGAAGAGAUAC 3′
	5′ GUAUCUCUUCAUAGCCUUA 3′
**qPCR primers**	**Sequence**
**SRSF1 (SF2)**	5′ CATCGACCTCAAGAATCGCC 3′
	5′ CCCATCGTAATCATAGCCGT 3′
**SRSF2 (SC35)**	5′ TTCGCCTTCGTTCGCTTTC 3′
	5′ CCGTAGCGCGCCATTTGCA 3′
**SRSF3 (SRp20)**	5′ TGCTTTTGTTGAATTTGAAGA 3′
	5′ CATTCGACAGTTCCACTCTT 3′
**SRSF5 (SRp40)**	5′ AATGATAGACGAAATGCTCC 3′
	5′ CCGCAAACGTTACTTCCCC 3′
**SRSF7 (9G8)**	5′ GGGCTTTCAGTTATTATGGT 3′
	5′ GGGCAGGTGGTCTATCAAAA 3′
**SRSF9 (SRp30c)**	5′ CTGGGGATGTCTGTTATGCT 3′
	5′ TGAGAGCGGAATTTGGTGT 3′
**Tra2β**	5′ TTGATGGGCGTAGGATCAGAGTTG 3′
	5′ TCCTCTGTCATAGTAATCCCGACG 3′
**GAPDH**	5′ TGCCAAATATGATGACATCAAGAA 3′
	5′ GGAGTGGGTGTCGCTGTTG 3′

(A)

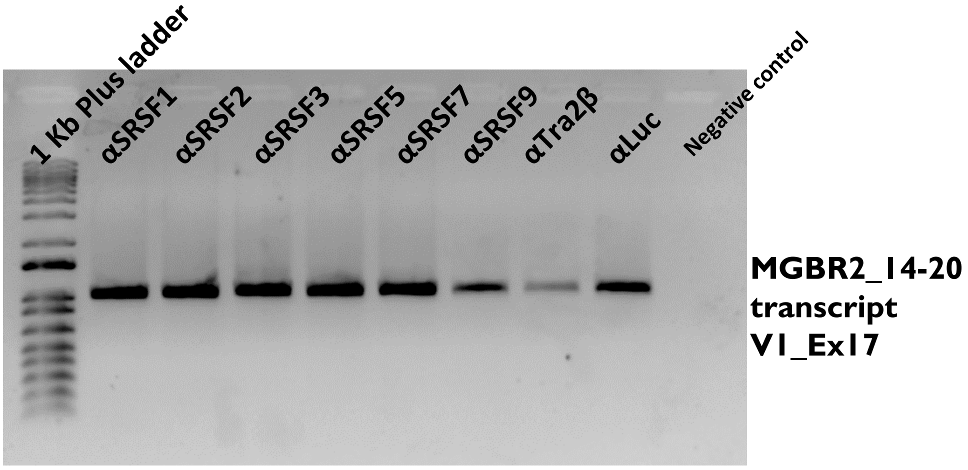

(B)

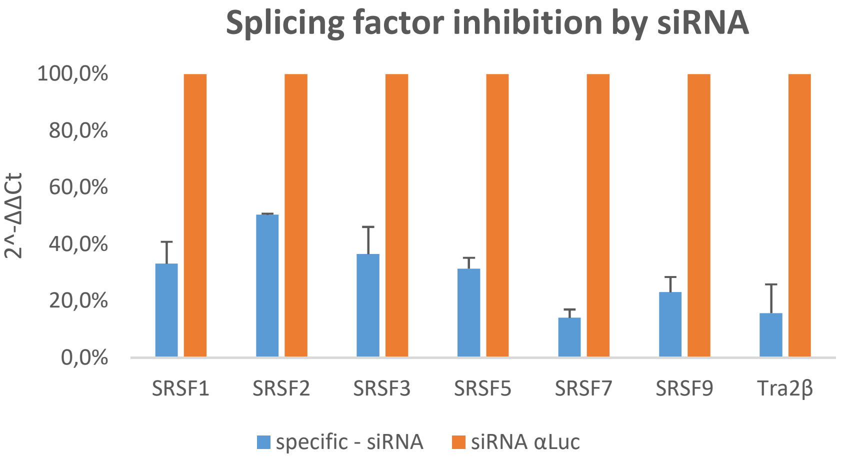

Supplemental Figure S1. Regulation study of exons 14 and 15. A) Impact of siRNA inhibition of splicing factors on minigene transcript. Total RNA was purified from MCF-7 transfected cells and cDNA was amplified with pMAD607-FW and RTBR2_EX17RV[2] as described in Materials and Methods. B) Inhibition of specific splicing factors was monitored by real-time quantitative PCR (RT-qPCR). Expression data is shown relative to the siRNA control against luciferase.

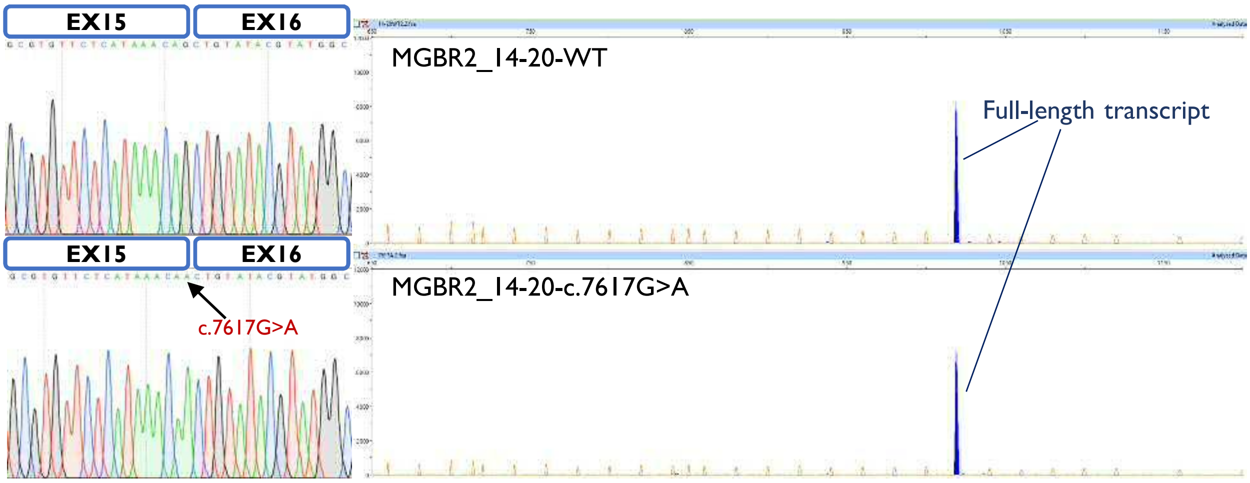

Supplemental Figure S2. Functional assay of variant c.7617G>A that affects the last nucleotide of exon 15. On the right, sequences of transcripts generated by the wt minigene and by the c.7617G>A minigene. On the left, fragment analysis of RT-PCR products are shown. Blue peaks show the FAM-labelled PCR product and orange peaks are the LIZ1200 size standard.
